# Draft Genome Sequence of Rhodococcus erythropolis HX7, a Psychrotolerant Soil-Derived Oil Degrader

**DOI:** 10.1128/MRA.01353-20

**Published:** 2021-01-21

**Authors:** Andrey D. Novikov, Konstantin V. Lavrov, Artem S. Kasianov, Aleksei A. Korzhenkov, Tatyana A. Gubanova, Alexander S. Yanenko

**Affiliations:** a NRC Kurchatov Institute, Moscow, Russia; b NRC Kurchatov Institute-GOSNIIGENETIKA, Kurchatov Genomic Center, Moscow, Russia; c Vavilov Institute of General Genetics, Moscow, Russia; University of Southern California

## Abstract

We describe here a 6.6-Mb draft genome sequence of Rhodococcus erythropolis strain HX7, which was obtained from soil samples collected from the northern Arkhangelsk region in the Russian Federation. This genomic resource will support further study of mechanisms of cold-resistant oil degradation in soil and potentially aid in soil bioremediation in cold oil-producing regions.

## ANNOUNCEMENT

Many terrestrial oil production sites are located in the cold regions of Earth. Oil production is accompanied by oil pollution, which leads to accumulation of oil in soil (see, for example, reference 1). Cleansing the oil from soil in cold conditions can be intensified with specific bacteria, which should be able to grow in the cold and metabolize oil (2).

**FIG 1:**
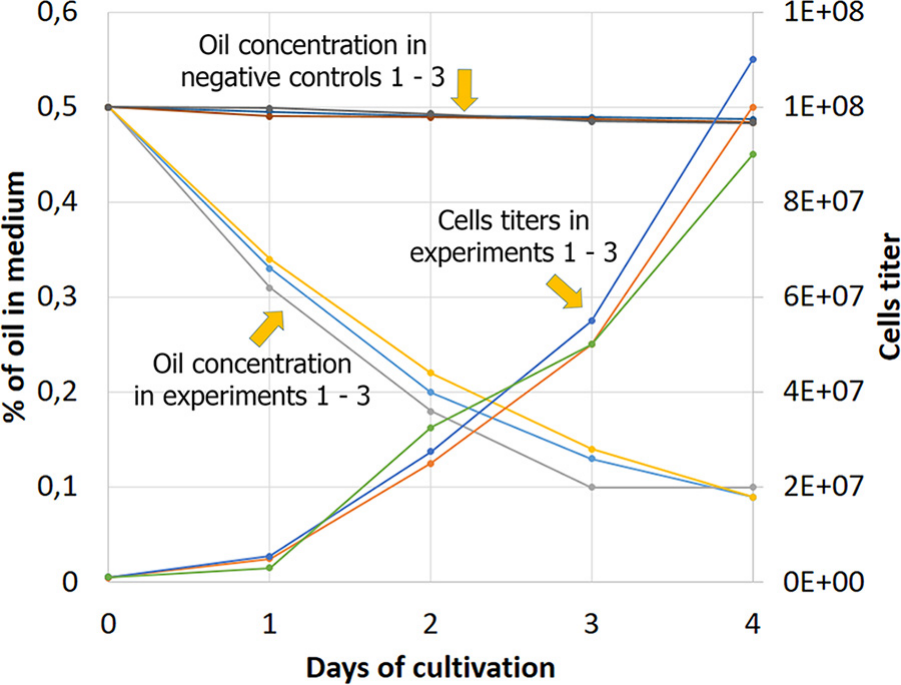
Growth of R. erythropolis strain HX7 on minimal medium with oil as a sole source of carbon (cell titers 1 to 3) and oil degradation during growth (oil concentrations 1 to 3). The experiment was conducted in triplicate, and individual values of every test are presented, including oil concentrations in blank tests without added R. erythropolis HX7 cells (negative controls 1 to 3). Oil degradation is presented as the percentage of residual oil, which was measured using a gravimetric method after chloroform extraction. Cell titers were measured by counting CFU after spreading the dilutions of cultures onto agar plates with minimal medium (see text) with glucose as the carbon source instead of oil.

Here, we report the genomic data of the oil-degrading strain Rhodococcus erythropolis HX7, which was isolated from oil-polluted soil near Naryan-Mar in the Arkhangelsk region of the Russian Federation (64.539393, 40.516939). Minimal medium of the following composition was used for isolation and evaluation of the strain: 1 g/liter KNO_3_, 0.6 g/liter KH_2_PO_4_, 1.4 g/liter Na_2_HPO_4_, 0.3 g/liter MgSO_4_, oil 0.5%, pH 7.2, and agar-agar added if needed (20 g/liter). The oil used was degassed, medium, paraffin-naphthenic oil of the West Siberian field. Oil was presterilized at 50.6 mPa and 110°C for 20 min and added separately to each batch of solid medium or each flask with liquid medium. Soil extracts were spread onto agar plates, and a group of colony-forming strains was selected. Among them, strain HX7 was selected due to its maximal growth speed at 5°C in liquid medium in Erlenmeyer flasks (100 ml of medium, shaking at 300 rpm, in a dark room) and for its ability to degrade oil (Fig. 1).

The genomic DNA was extracted from the cells using the phenol-chloroform method, and a DNA fragment library was prepared with a KAPA HyperPlus kit according to the manufacturer’s recommendations. Sequencing was performed on an Illumina MiSeq system at the NRC Kurchatov Institute (Moscow, Russia), and 1,434,670 paired-end reads with an average 251-bp length were generated. During read processing and genome assembling, default parameters were used for all software unless otherwise specified. Preassembly quality control of reads was done using FastQC version 0.11.9 (https://www.bioinformatics.babraham.ac.uk/projects/fastqc/). Reads were trimmed based on quality (qtrim=r, trimq=18), adapter sequences were removed with BBduk, and overlapping reads were merged with BBmerge from BBMap; all BB programs were version 38.75 (https://sourceforge.net/projects/bbmap/). Then, reads were assembled using SPAdes (3) version 3.14 with the “careful” option, producing 75 contigs with a total genome size of 6,567,540 bp (GC content, 62.4%; *N*_50_, 774,745 bp) and an average 105-fold coverage.

Automatic annotation was performed using the NCBI Prokaryotic Genome Annotation Pipeline (PGAP) version 4.13, generating a total of 6,156 genes, 6,020 potentially protein-coding genes, 73 genes of noncoding RNA, including 52 tRNA genes and 21 rRNA genes, 63 pseudogenes, and 1 CRISPR array. An analysis of similarity among *Rhodococcus* genomes, measured as average nucleotide identity (ANI), shows that the genome of R. erythropolis HX7 is most similar to that of R. erythropolis NBRC 15567 (GenBank accession number GCA_001552595.1), with an ANI of 98.73%. The ANI was calculated using ani.rb script (https://github.com/lmrodriguezr/enveomics/blob/master/Scripts/ani.rb) with default settings.

Improvement of the effectiveness of bioremediation methods could be achieved by using genetically engineered strains, which are often more enzymatically active than naturally occurring pollutant-degrading strains (4). The genome information for strain HX7 will aid in the construction of genetically engineered strains for not only bioremediation but also other *Rhodococcus*-based biotechnologies, for example, using the strain as a host for overexpression of biotechnologically relevant enzymes (5, 6).

### Data availability.

The genome information has been deposited at NCBI GenBank; the raw sequencing reads are available under SRA number SRX9614685, and the draft genome sequence is available under GenBank accession number JADOEI000000000.1. The version of the genome described in this paper is the first version.
